# MARM: a framework for malignancy risk prediction from host-derived CNV in bronchoalveolar lavage fluid mNGS data with microbial admixture

**DOI:** 10.3389/fmicb.2026.1846545

**Published:** 2026-05-21

**Authors:** Zhili Chang, Xiaonan Wang, Minchao Zhao, Xian Zhang, Shuotong Li, Yuxuan Liu, Shuqun Zhang, Jiayin Wang, Xuwen Wang

**Affiliations:** 1 School of Computer Science and Technology, Xi'an Jiaotong University, Xi'an, China; 2 Shaanxi Engineering Research Center of Medical and Health Big Data, Xi'an Jiaotong University, Xi'an, China; 3 Medical Department, Nanjing Geneseeq Technology Inc., Nanjing, China; 4 Shenzhen University, Shenzhen, China; 5 Nanjing Hankai Academy, Nanjing, China; 6 The Comprehensive Breast Care Center, The Second Affiliated Hospital of Xi'an Jiaotong University, Xi'an, China

**Keywords:** heterogeneous labels, host-derived copy number variation, machine learning, malignancy prediction, metagenomic next-generation sequencing, microbial admixture

## Abstract

Early identification and risk assessment of malignancy are essential for improving clinical decision-making and patient outcomes. Bronchoalveolar lavage fluid (BALF) metagenomic next-generation sequencing (mNGS) data contain both microbial and host-derived signals, and a key challenge in extending such data to tumor-associated applications is the robust extraction of host features with discriminative value for malignancy from this complex, admixed background. To address this problem, we developed MARM, a malignancy risk prediction method centered on host-derived copy number variation (CNV). Using host-derived reads from BALF mNGS data, MARM performs genome-wide window-based coverage quantification, normalization and bias correction, reference baseline construction, and principal component-based denoising to derive window-level CNV features for malignancy risk modeling. In addition, a pseudo-label-based extension strategy was introduced to incorporate weakly labeled samples through high-confidence screening, and the performance of XGBoost, Random Forest, and generalized linear models (GLM) was systematically evaluated using CNV features, microbial features, and combined features. Models built on host-derived CNV features consistently outperformed those based on microbial features and achieved performance comparable to combined-feature models, while joint modeling did not provide a stable additional benefit. These findings indicate that, under the current data setting and feature construction strategy, CNV represents a more stable and informative discriminative signal than microbial features. Among the evaluated classifiers, XGBoost showed the best compatibility with window-level CNV features and outperformed Random Forest and GLM overall. On the independent validation set, the pseudo-label-enhanced MARM achieved the best overall performance, with a sensitivity of 0.686, specificity of 0.975, accuracy of 0.847, and Youden index of 0.671. By contrast, microbial features did not show stable independent discriminative ability, and combined modeling did not yield clear or sustained performance gains. Together, these results indicate that, in microbially admixed BALF mNGS data, host-derived CNV is more suitable than the evaluated microbial features as the core modeling signal for malignancy risk prediction. MARM provides a new methodological framework for malignancy prediction in complex clinical samples and offers a reference for deeper exploitation of host-derived signals in mNGS data and related auxiliary diagnostic applications.

## 1 Introduction

Malignancy remains a major threat to human health, and its early detection and risk assessment are critical for optimizing clinical decision-making, improving treatment efficacy, and enhancing patient outcomes (Roychowdhury et al., 2011; Reuter et al., 2015; Lightbody et al., 2019; Garima et al., 2025). In the clinical management of pulmonary diseases, malignant tumors and infectious disorders often present with overlapping symptoms, nonspecific imaging findings, and inconsistent routine test results, making early identification and accurate stratification particularly challenging. Methods that can reliably extract malignancy-associated molecular signals from complex clinical specimens are therefore of practical importance for improving the auxiliary diagnosis of pulmonary malignancies (Roychowdhury et al., 2011; Reuter et al., 2015; Lightbody et al., 2019; Garima et al., 2025). mNGS was originally developed for rapid pathogen detection in infectious diseases, with the key advantage of unbiased identification of diverse microorganisms in clinical samples without requiring predefined targets (Simner et al., 2018; Gu et al., 2019; Duan et al., 2021). With broader clinical use, it has become clear that mNGS data contain not only pathogen-related information but also substantial host-derived sequence information, creating the possibility of simultaneous pathogen detection and host abnormality profiling (Gu et al., 2021; Guo et al., 2021; Hemmati et al., 2024). This feature is especially relevant for BALF samples, in which mNGS data typically include abundant microbial reads together with host-derived reads, generating a characteristic microbially admixed data background. Under these conditions, a central challenge in extending BALF mNGS beyond infectious disease testing toward tumor-associated applications is how to robustly extract host-derived signals with discriminative value for malignancy from this complex mixture (Gu et al., 2021; Guo et al., 2021; Hemmati et al., 2024).

Among the host-derived signals available in this setting, CNV is one of the most common genomic abnormalities in malignancy, and the associated chromosomal instability has been widely documented across both solid tumors and hematologic cancers (Shlien and Malkin, 2009; Kuiper et al., 2010). Coverage-based CNV detection methods can use host read-depth signals to identify large-scale copy number alterations (Fan et al., 2008; Yoon et al., 2009; Bianchi et al., 2015; Dharajiya et al., 2018; Ji et al., 2019). Previous studies have shown that host-derived sequence data generated by mNGS can be used to detect CNV signals and thereby assist in identifying occult malignant lesions (Gu et al., 2021; Guo et al., 2021; Huang et al., 2023; Hemmati et al., 2024). CNV analysis in body fluids, BALF, and tissue samples has demonstrated value for tumor-associated auxiliary diagnosis, particularly in patients whose clinical presentation overlaps between infection and malignancy and in whom routine examinations fail to establish a clear diagnosis (Gu et al., 2021; Guo et al., 2021; Huang et al., 2023; Hemmati et al., 2024; Li et al., 2025). For example, Gu et al. reported no false-positive findings in a negative control cohort of body fluid samples, suggesting high specificity for this approach; notably, CNV signals were still detectable in some cases that were negative by conventional cytology or flow cytometry but were ultimately confirmed as malignant (Gu et al., 2021). Huang et al. likewise showed in patients with lower respiratory tract disease that mNGS-based concurrent pathogen detection and CNV analysis could facilitate the recognition of occult tumors, and that a substantial proportion of CNV-positive cases had initially been misdiagnosed as non-neoplastic disease (Huang et al., 2023). Together, these findings suggest that, in microbially admixed BALF mNGS data, host-derived CNV may represent a practically useful malignancy-associated signal.

By contrast, although previous studies have suggested that microbiome-related features may be associated with the development and progression of certain malignancies (Liu and Zhang, 2022; Xue et al., 2023), and that the structure and diversity of the lower respiratory tract microbiota may be altered in patients with lung cancer (Pizzo et al., 2022; Su et al., 2023), microbial features are also more susceptible to confounding from infection status, local colonization, antibiotic exposure, and sampling variation. Their stability for malignancy risk prediction at the individual level therefore remains uncertain (Liu and Zhang, 2022; Pizzo et al., 2022; Su et al., 2023; Xue et al., 2023). In the clinically relevant setting of BALF mNGS, where microbial signals are highly abundant and intermixed with host-derived reads, building malignancy prediction models around host-derived CNV may thus represent a more direct and operationally practical strategy. However, existing studies have largely focused on the use of mNGS for pathogen detection or supportive CNV interpretation, and a more complete methodological framework for systematic modeling of host-derived CNV and malignancy risk prediction in microbially admixed BALF mNGS data is still lacking. At the same time, real-world clinical datasets commonly exhibit label heterogeneity: some samples are supported by pathology or definitive clinical diagnosis and can serve as high-quality labeled data, whereas others are only clinically suspected of malignancy or lack final diagnostic confirmation and therefore represent weakly labeled data. Restricting model development to high-quality labeled samples often limits sample size and disease-spectrum coverage, whereas directly incorporating weakly labeled samples may introduce label noise and model bias. Accordingly, when developing a host-CNV-based model for malignancy risk prediction, an additional practical methodological question is how to make effective use of heterogeneously labeled data to improve model performance.

**Figure 1:**
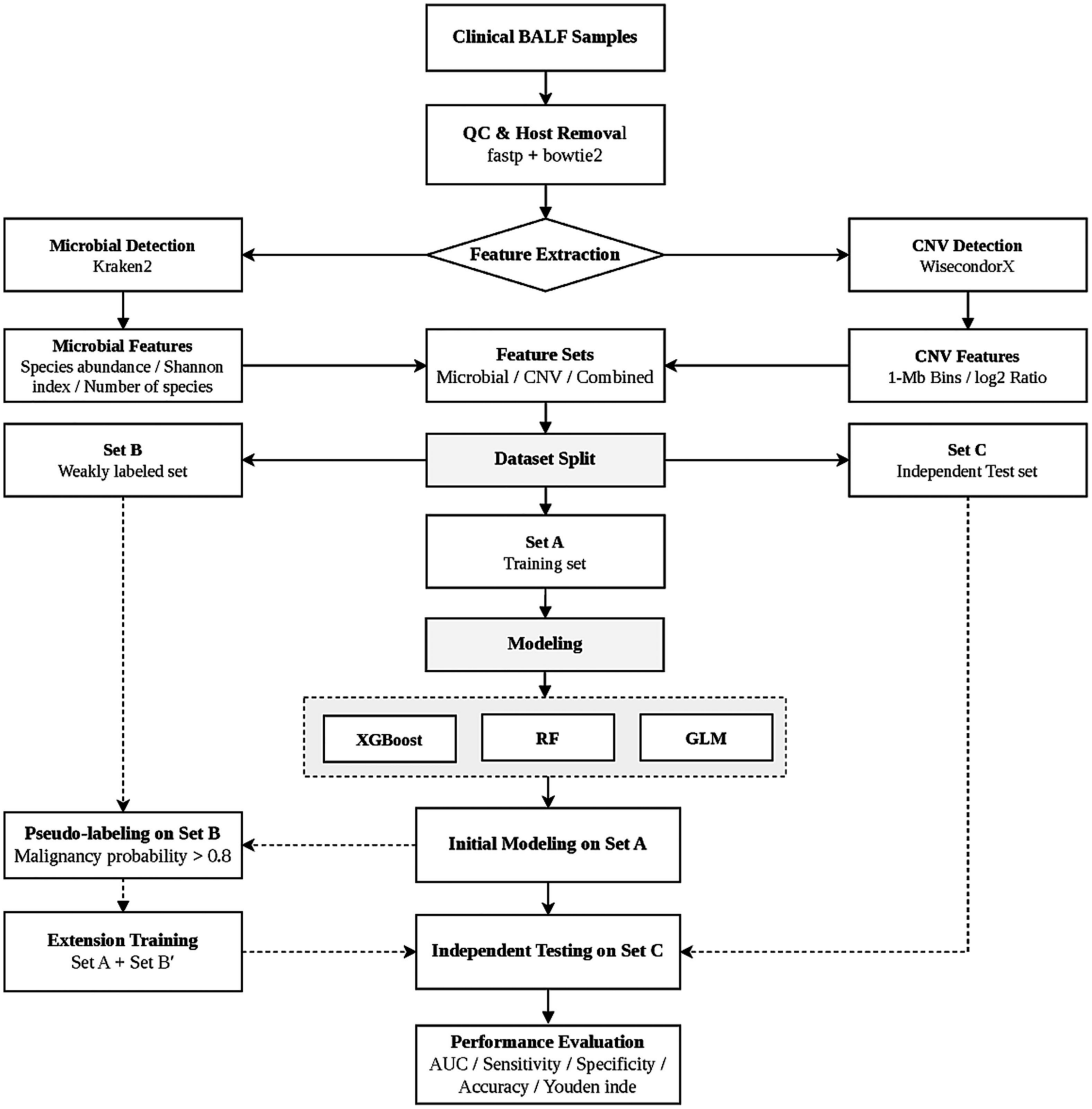
Schematic overview of the MARM workflow.

Here, we developed MARM, a malignancy risk prediction framework for microbially admixed BALF mNGS data, in which host-derived CNV serves as the central modeling signal. Starting from host-derived reads retained in BALF mNGS data, the framework performs genome-wide window-based coverage quantification, normalization and bias correction, reference baseline construction, and principal component-based denoising to derive window-level CNV features for downstream risk modeling. Given the high dimensionality, local sparsity, noise, and potential nonlinear structure of these features, XGBoost was adopted as the default implementation of the framework. In parallel, Random Forest and generalized linear models were included as comparative implementations to evaluate model adaptability under the same task setting. In addition, to address the common issue of label heterogeneity in real-world clinical datasets, we incorporated a pseudo-label-based extension strategy to make controlled use of weakly labeled samples and to test whether such samples could provide additional predictive value without disrupting the core modeling structure. Through this work, we sought to establish a practical method for malignancy risk prediction in microbially admixed BALF mNGS data and to provide a methodological reference for the extraction, representation, and utilization of host-derived signals in this type of clinical sequencing dataset. A schematic overview of the overall analytical workflow is provided in Figure 1.

## 2 Materials and methods

### 2.1 BALF samples

This study included mNGS data generated from BALF samples. All samples were sequenced on the MGI200 platform using single-end 50-bp reads. Because BALF mNGS data contain both microbial and host-derived sequences, they represent a microbially admixed sequencing background. The present study focused on this type of data and extracted host-derived CNV signals for malignancy risk modeling. To ensure the reliability of host-derived CNV estimation from shallow BALF mNGS data, additional inclusion criteria were applied before CNV feature extraction. Samples were required to contain at least 5 Mb of host-derived reads and to achieve an effective host-genome coverage of no less than 0.05×. Samples failing to meet these criteria were excluded from downstream CNV-based modeling. Sample-level sequencing quality-control statistics, including total raw reads, high-quality reads and their proportion, Q30, duplication rate, host-derived read counts, and host-read ratio, are summarized in Supplementary Table S1. According to label quality and model-development purpose, all samples were divided into three datasets. Set A served as the high-quality labeled training set and consisted of 887 samples with definitive diagnoses, including 243 malignant and 644 non-malignant cases. This dataset was used for baseline model development, including internal model training, hyperparameter selection, and five-fold cross-validation. Set B was a weakly labeled sample set comprising 1,412 cases, including 778 samples without explicit tumor labels and 634 samples clinically suspected of malignancy. This dataset was not used for initial supervised training, but was reserved for subsequent pseudo-label selection and extension training. Set C served as an independent validation set and included 186 samples, comprising 86 malignant and 100 non-malignant cases, for final model evaluation. The overall analytical workflow consisted of three stages. First, host-derived CNV features were extracted from Set A, and baseline models were developed using five-fold cross-validation for internal training and parameter selection. Second, the baseline model was applied to Set B, from which high-confidence pseudo-labeled samples were selected for extension training. Third, both the baseline models and the pseudo-label-extended models were evaluated on Set C, which was reserved throughout the study as a fully independent test set. Microbial features were extracted from the same mNGS data and used for supplementary analyses.

### 2.2 Microbial identification and construction of the microbial feature matrix

Raw sequencing data in FASTQ format were processed with fastp v0.23.4 (Chen et al., 2018) for quality control, including adapter trimming, low-quality base filtering, and length filtering, in order to minimize the impact of sequencing errors and low-quality reads on downstream taxonomic classification. The quality-filtered reads were then aligned to the human reference genome hs37d5 using bowtie2 v2.5.1 (Langmead and Salzberg, 2012) in end-to-end mode to remove host-derived sequences. Unmapped reads were retained for subsequent microbial identification. Microbial classification was performed using Kraken2 v2.1.3 (Wood et al., 2019). The Kraken2 reference database was built in-house from reference genomes obtained from GenBank (release 202404) (Clark et al., 2015) and covered four major microbial groups: bacteria, fungi, parasites, and viruses. Classification results were summarized at the species level, and the number of assigned reads was counted for each detected microbial species in each sample. Because low-abundance microbial detections in BALF mNGS data may be influenced by background contamination, stochastic sampling effects, and limited read misassignment, a species was considered positively detected only when its read count exceeded 2 in a given sample. Before model construction, low-frequency species were further filtered. As most rarely detected species appeared in only a small subset of samples, including them directly in modeling would increase feature dimensionality, exacerbate sparsity, and introduce additional random noise. Therefore, only species meeting the positive-detection criterion in at least 10% of samples were retained for downstream analysis as part of the prespecified microbial feature-construction pipeline. This yielded a sample-by-species microbial feature matrix, in which each row represented a BALF sample, each column represented a retained microbial species, and each matrix entry corresponded to the number of reads assigned to that species in the given sample. This matrix was used to characterize microbial detection patterns and relative abundance differences across BALF samples, and served as the input for the microbial-only and combined-feature models. To assess whether model performance was sensitive to the species-retention threshold, we further performed a supplementary sensitivity analysis by varying the prevalence cutoff from 1 to 5 and 10% while keeping the remaining modeling workflow unchanged. This analysis was intended to examine whether inclusion of lower-frequency microbial species could improve predictive performance or instead introduce additional sparsity and noise. In addition, microbial features in the present study were defined primarily at the species-detection and abundance level and were not further stratified according to pathogenic potential, commensal status, or likely environmental contamination.

### 2.3 Identification of host-derived CNV features

Host-derived CNV features were extracted from microbially admixed BALF mNGS data for subsequent malignancy risk modeling. After basic quality control, sequencing reads were realigned to the human reference genome hs37d5, and host-aligned reads were retained for CNV analysis. Because BALF mNGS data contain large amounts of both microbial and host-derived sequences, while the overall sequencing depth is relatively limited, single-locus variation signals are insufficient to robustly reflect genome-wide copy number status. We therefore adopted a window-based coverage strategy to represent host-derived CNV and to capture more stable chromosomal-level abnormality signals. Window-level CNV analysis was performed using WisecondorX v1.2.9 (Raman et al., 2019). Across the whole genome, non-overlapping 1-Mb windows were defined, yielding a total of 3,113 bins, and host-derived read counts were calculated for each bin in each sample. This step transformed raw host alignment signals into a genome-wide window-level coverage matrix that could be compared across samples and used for subsequent normalization, correction, and abnormality scoring. Because GC content, mappability, and baseline coverage differ across genomic windows, directly using raw read counts would introduce technical bias. Accordingly, window-level read counts were first normalized and then corrected for GC content and mappability to reduce the influence of non-biological factors on coverage profiles. After normalization and bias correction, a copy number ratio (CNR) was calculated for each genomic window relative to the reference baseline and then converted to a log2 ratio. The log2 ratio was used to characterize the direction and magnitude of copy number deviation relative to the reference state, with positive values indicating relative copy-number gain and negative values indicating relative copy-number loss. Compared with untransformed read counts, log2 ratios are more suitable for between-window and between-sample comparisons and facilitate subsequent quantification of abnormality strength.

To define the background coverage distribution under non-malignant conditions, a reference baseline was constructed using 200 non-malignant BALF samples and was then used consistently throughout CNV feature derivation. This baseline was used to describe the range of window-level coverage variation under host-normal conditions and to support subsequent normalization and abnormality scoring. Because BALF mNGS represents shallow sequencing and host-read fractions, total sequencing depth, and batch conditions may vary across samples, window-level coverage profiles can contain substantial systematic background fluctuation. To reduce such shared noise components, principal component analysis (PCA) was further performed on the non-malignant reference set to remove common background variation attributable to sequencing depth, batch effects, and other non-biological factors, thereby improving the separability of true CNV-related signals. In this study, this denoising step was treated as part of the fixed CNV preprocessing workflow and was not varied across the downstream model comparisons. Following PCA denoising, a Z score was calculated for each genomic window and used as the input feature for downstream classification models according to the following equation:


$$Z=\frac{x-\mu }{\sigma }$$

where 𝑥 denotes the log2 ratio of a given genomic window in the test sample, 𝜇 denotes the mean log2 ratio of the corresponding window across the reference baseline samples, and 𝜎 denotes the corresponding standard deviation. The Z score reflects the standardized deviation of a given window in the test sample relative to the non-malignant background distribution. A larger absolute Z score indicates a greater deviation from the background copy-number level. Compared with raw read counts, corrected coverage, or unstandardized log2 ratios, Z scores provide a unified measure of relative abnormality intensity across genomic regions and are therefore more suitable as input features for cross-sample comparison and machine-learning-based modeling. Because host-read fractions and total sequencing depth varied across BALF mNGS samples, not all 3,113 bins were stably covered in all samples. We therefore applied an additional feature-availability filter. Any bin that was available in fewer than 25% of samples was considered unstable and removed to reduce the impact of low-coverage windows on model training. After filtering, 2,523 window-level CNV features were retained. Each sample was thus represented by a CNV feature vector composed of the Z scores from these 2,523 1-Mb bins, capturing genome-wide chromosomal instability and large-scale copy-number gain/loss patterns for subsequent malignancy risk prediction.

### 2.4 Construction of malignancy prediction models

We developed MARM, a malignancy prediction framework for microbially admixed BALF mNGS data, with malignancy versus non-malignancy as the binary classification task. The core of MARM is the extraction of host-derived CNV signals from a microbially admixed sequencing background and their representation as window-level feature vectors for downstream classification. The framework emphasizes robust extraction, standardized representation, and risk modeling of host-derived CNV from BALF mNGS data; within this framework, the specific machine-learning classifier can be adapted according to the study objective, sample characteristics, and data scale. Given that the window-level CNV Z-score features used in this study are high-dimensional, locally sparse, noisy, and potentially governed by complex nonlinear relationships, the classifier must be able to identify abnormal patterns relevant to malignancy while maintaining adequate generalizability under noisy conditions. On this basis, extreme gradient boosting (XGBoost) was selected as the default implementation. XGBoost is an ensemble learning method based on gradient-boosted decision trees that can iteratively combine weak learners to capture complex nonlinear relationships and feature interactions. For the CNV features considered here, abnormalities in different genomic windows are unlikely to act independently; rather, they jointly reflect underlying chromosomal instability. XGBoost is well suited to learning such nonlinear patterns in high-dimensional feature space and performs implicit feature selection during tree splitting. In addition, its built-in regularization helps reduce the influence of noisy and redundant features during model fitting, making it well suited to the mNGS-derived feature data used in this study. Accordingly, XGBoost models were first constructed using host-derived CNV features, microbial features, and their combination. Among these, the XGBoost model based on CNV features served as the core implementation of the present study, with the 2,523-dimensional window-level CNV Z-score vector as input. Microbial-only and combined-feature models were included as supplementary analyses to evaluate the modeling value of microbial information and multi-source feature integration in this task.

To further assess whether the default implementation of MARM was well matched to the present data, Random Forest and GLM were also constructed as comparator models, representing a tree-based ensemble comparator and a linear baseline model, respectively. All models were first trained on Set A to establish baseline predictors, and their performance in malignancy prediction was then compared under a unified development-validation workflow. Accordingly, model comparison in the present study was based on a fixed independent validation design rather than on repeated k-fold cross-validation.

### 2.5 Model training and hyperparameter settings

All model training, internal validation, hyperparameter tuning, and downstream performance evaluation were performed in a unified framework in R 4.5.0. To ensure reproducibility and comparability across classifiers, Random Forest, XGBoost, and generalized linear models (GLM) were all implemented using the caret package under a consistent binary-classification setting for malignancy versus non-malignancy. Class probability output was enabled for all models, and model performance during training was summarized using twoClassSummary, with AUROC as the primary optimization metric.

For baseline model development, Set A was used as the high-quality labeled dataset for internal training and parameter selection. In this stage, five-fold cross-validation was applied to evaluate baseline model robustness and to tune model parameters where applicable. This internal validation step was intended to distinguish the stability of baseline model development from the subsequent pseudo-label-based extension procedure. Set C was not involved in model training or parameter tuning at any stage and was reserved exclusively for final independent evaluation.

Specifically, Random Forest was implemented using the “rf” method, with the number of trees fixed at 500 and mtry tuned through the default caret search grid. XGBoost was implemented using the “xgbTree” method, and the default caret tuning grid was used to search over nrounds, max_depth, eta, gamma, colsample_bytree, min_child_weight, and subsample, with nthread set to 1 during training. GLM was implemented using the “glm” method with family = “binomial” and did not involve additional hyperparameter tuning.

For retraining after pseudo-label expansion, that is, when Set A was combined with the filtered pseudo-labeled subset Set B′, cross-validation was not repeated. Instead, models were refit using fixed parameter settings to ensure a deterministic and reproducible extension-training procedure. Under this setting, Random Forest was trained with ntree = 500 and mtry = floor(sqrt(p)), where p denotes the total number of input features. XGBoost was trained with fixed parameters of nrounds = 100, max_depth = 6, eta = 0.3, gamma = 0, colsample_bytree = 1.0, min_child_weight = 1, and subsample = 1.0. GLM was refit using family = “binomial.”

On the basis of this conventionally validated baseline modeling procedure, weakly labeled samples in Set B were incorporated only as a subsequent enhancement step through controlled pseudo-label-based extension training, rather than as a substitute for standard internal validation.

### 2.6 Pseudo-label-based extension training with weakly labeled samples

To further leverage the information contained in weakly labeled real-world clinical samples, we introduced a pseudo-label-based extension training strategy within the baseline risk prediction framework. This strategy was built on the principle of prioritizing high-quality labeled samples for initial model development: a baseline prediction model was first established using Set A, and information from the weakly labeled Set B was then incorporated in a controlled manner. The purpose was to expand the effective sample pool while minimizing the direct impact of label noise on model training, and to assess whether weakly labeled samples could provide additional predictive value for malignancy risk prediction. Although Set B samples contained some clinical context, they lacked definitive gold-standard diagnostic labels. Directly incorporating them into supervised training could therefore propagate labeling errors, blur class boundaries, and increase model bias. For this reason, Set B was not used in the initial supervised training stage. Instead, it was incorporated indirectly through pseudo-label-based extension training. The basic idea of this strategy was to train a baseline model on the high-quality labeled samples, apply that model to the weakly labeled samples, assign pseudo-labels only to samples with highly consistent predictions, and then incorporate those samples into an extended training procedure. In this way, potentially informative weakly labeled cases could be utilized while controlling the risk of mislabeling.

Specifically, the default baseline model was first trained on Set A and then applied to Set B on a sample-by-sample basis to generate malignancy prediction probabilities. Because the central task of this study was malignancy risk prediction, and because potentially malignant cases in the weakly labeled set were expected to provide more informative additions to the positive class, only samples predicted as malignant with a prediction probability greater than 0.8 were selected as high-confidence pseudo-labeled samples. To assess the sensitivity of the pseudo-label extension strategy to the confidence threshold, we further compared thresholds of 0.7, 0.8, and 0.9 while keeping the remaining modeling workflow unchanged. This analysis was designed to evaluate whether the choice of threshold would materially affect the performance of the extended MARM model and to determine an appropriate balance between pseudo-label purity and sample expansion. These selected samples were assigned pseudo-labels and incorporated into the extended training set together with the high-quality labeled samples from Set A for model retraining. The purpose of this threshold was to preferentially retain weakly labeled samples with high predictive confidence and thereby reduce noise accumulation and error propagation arising from the inclusion of low-confidence pseudo-labeled samples. To evaluate the practical impact of this strategy, two classes of models were constructed: baseline models trained using Set A alone, and extended models retrained by incorporating pseudo-labeled samples selected from Set B. Their performances were then compared on the independent validation set, Set C, to determine whether pseudo-label-based extension training could improve classification performance on unseen data. This analysis was intended to test whether the incorporation of weakly labeled samples could provide additional useful information within the malignancy prediction framework proposed here.

### 2.7 Model evaluation and statistical analysis

Model training, performance evaluation, and visualization were all performed in R 4.5.0. To comprehensively assess classification performance in the malignancy prediction task, we used sensitivity, specificity, accuracy, the Youden index, and the area under the receiver operating characteristic curve (AUC) as the primary evaluation metrics. Sensitivity was used to quantify the ability of a model to identify malignant samples, specificity to quantify the ability to exclude non-malignant samples, accuracy to measure overall classification correctness, the Youden index to summarize the balance between sensitivity and specificity, and AUC to assess overall discriminative performance. All models were evaluated in a unified manner on the independent validation set, Set C, to ensure comparability across different feature inputs, modeling methods, and training strategies. Thus, preprocessing and model comparison were conducted within a fixed development-validation framework, with final performance assessed only on Set C. Performance comparisons included both baseline models trained using Set A alone and extended models retrained after incorporating pseudo-labeled samples from Set B. By comparing model performance on Set C under this unified evaluation scheme, we assessed the effects of feature source, modeling method, and pseudo-label-based extension training on malignancy prediction performance. In addition to point estimates, 95% confidence intervals were reported for sensitivity, specificity, and accuracy on the independent validation set to better reflect uncertainty in model performance estimates. Particular emphasis was placed on the core models constructed from host-derived CNV features, as well as on whether pseudo-label-based extension training produced measurable performance gains. Microbial-only and combined-feature models were treated as supplementary analyses for comparing the contributions of different information sources to this prediction task.

## 3 Results

### 3.1 Baseline characteristics and microbiome-related metrics

The composition of the three datasets has been described in the Materials and Methods section, and a further comparison of baseline characteristics is shown in Table 1. In Set A, patients in the malignant group were older and more likely to be male than those in the non-malignant group, with both differences reaching statistical significance (both *p* < 0.001). In Set B, no significant age difference was observed between the unlabeled group and the clinically suspected malignancy group, whereas sex distribution differed significantly (*p* = 0.0017). In Set C, neither age nor sex distribution differed significantly between the malignant and non-malignant groups. Further examination of microbiome-related metrics showed that their patterns were not consistent across datasets. In Set A, the malignant group had a higher Shannon index and a greater number of microbial species than the non-malignant group (both *p* < 0.001), and also showed a higher proportion of fungal infection (*p* = 0.0386). In Set B, the clinically suspected malignancy group had a higher Shannon index but a lower number of microbial species than the unlabeled group (both *p* < 0.001). In contrast, in Set C, no significant differences were observed between the malignant and non-malignant groups in either the Shannon index or the number of microbial species. Overall, microbiome-related metrics did not show a stable or consistent pattern across datasets, and in particular did not demonstrate clear discriminative ability in the independent validation set, Set C. This finding suggests that microbial signals were relatively unstable in the present dataset and provides context for the limited overall performance of microbiome-based models in subsequent analyses.

**Table 1:** Comparison of baseline characteristics and microbe-related metrics across datasets.

Dataset	Group	*n*	Age (years, mean ± SD)	Female, *n* (%)	Shannon index (mean ± SD)	Number of microbial species (mean ± SD)
Set A	Malignant tumor	243	64.29 ± 11.25	63 (25.9)	4.32 ± 1.35	374.56 ± 241.46
Set A	Non-malignant tumor	644	56.90 ± 15.84	278 (43.2)	3.69 ± 1.58	293.24 ± 212.03
Set B	Unknown	778	60.91 ± 19.30	261 (33.5)	2.92 ± 1.33	341.02 ± 196.63
Set B	Suspected malignant tumor	634	61.27 ± 15.73	163 (25.7)	3.57 ± 1.50	293.14 ± 223.09
Set C	Malignant tumor	86	63.51 ± 14.22	17 (19.8)	3.34 ± 1.22	294.27 ± 172.53
Set C	Non-malignant tumor	100	61.47 ± 17.24	33 (33.0)	2.99 ± 1.17	339.52 ± 201.34

Only the major baseline variables are presented in this table.

### 3.2 Five-fold cross-validation performance of baseline models on set a

To distinguish the internal robustness of the baseline models from the additional effect of pseudo-label-based extension training, we first evaluated model performance on Set A using five-fold cross-validation. Overall, XGBoost showed the best internal performance under both CNV and combined-feature settings, whereas microbiome-only models remained weak across classifiers. Importantly, the relative ranking of different methods and feature types observed in cross-validation was consistent with the independent validation results on Set C, supporting the robustness of the baseline modeling strategy. The distribution of fold-wise AUC values is shown in Supplementary Figure S2.

### 3.3 Human-derived CNV is the core discriminative signal underlying MARM

**Figure 2:**
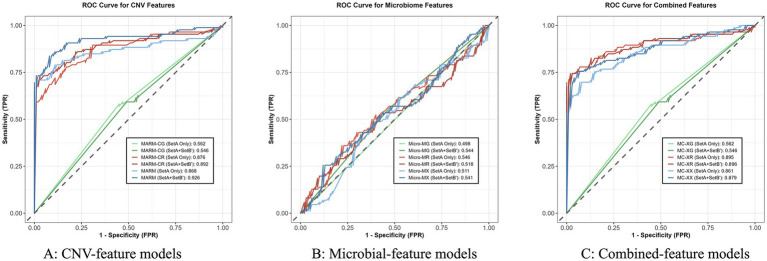
Comparison of ROC curves for different feature types across three machine learning methods. The figure presents ROC curves for models trained using Set A alone and using Set A plus pseudo-labeled Set B′. **(A)** CNV-feature models. **(B)** Microbial-feature models. **(C)** Combined-feature models.

The ROC curves for models built with different feature types are shown in Figure 2, and the corresponding overall performance metrics with 95% confidence intervals are summarized in Table 2. Overall, models based on host-derived CNV features achieved the best performance, whereas models based on microbial features showed consistently weak discriminative ability. Models combining both feature types performed similarly to CNV-based models, but did not demonstrate a stable additional benefit. This pattern was largely consistent across different modeling approaches, indicating that CNV features provided more robust discriminative value at both the cohort and model levels. Using Set A alone for training as an example, the XGBoost and Random Forest models based on CNV features achieved Youden indices of 0.642 and 0.587, respectively, whereas the corresponding microbial-feature models yielded values of only 0.046 and 0.043. After pseudo-label-based training expansion, the overall performance of the microbial models remained limited. For example, the Youden index of the XGBoost model based on microbial features decreased from 0.046 to 0.030, and that of the Random Forest microbial model declined from 0.043 to 0.017. These results indicate that, for malignant tumor risk prediction in this study, microbial features did not provide stable discriminative value under the current species-level representation, whereas the primary informative signal originated from host-derived CNV features. The combined-feature models performed similarly to the CNV-only models overall, but did not show a clear or consistent performance gain. For instance, within the XGBoost framework, when trained using Set A alone, the combined model achieved a Youden index of 0.632, slightly lower than the 0.642 obtained by the CNV-only model. After pseudo-label-based training expansion, the Youden index of the XGBoost combined model increased to 0.671, which was identical to that of the MARM model. Similarly, under the Random Forest framework, the combined models showed performance comparable to that of the CNV-only models, but did not exhibit a stable trend of outperforming CNV-based modeling alone. These findings suggest that, under the current data conditions, directly combining microbial features with CNV features did not provide clear additional discriminative value. Taken together, these results show that in microbially admixed BALF mNGS data, host-derived CNV features can more stably capture discriminative information associated with malignant tumors, whereas the evaluated microbial features functioned mainly as an unstable supplementary source of information. At the feature level, therefore, the use of host-derived CNV as the core modeling signal is a key basis for the effectiveness of MARM. We further examined whether the limited performance of microbial-feature models was attributable to the species-retention threshold. Under otherwise identical modeling procedures, prevalence cutoffs of 1, 5, and 10% yielded only minor differences in AUC across microbial models. Specifically, the AUCs of Micro-MG were 0.533, 0.510, and 0.544, respectively; those of Micro-MR were 0.516, 0.519, and 0.519; and those of Micro-MX were 0.539, 0.539, and 0.541. Overall, lowering the threshold to include more low-frequency species did not improve predictive performance, supporting the use of the 10% threshold in the main analysis. The detailed comparison is provided in Supplementary Figure S1.

**Table 2:** Performance comparison across different models, feature types, and training strategies.

Method	Feature	Strategy	Sensitivity (95% CI)	Specificity (95% CI)	Accuracy (95% CI)	Youden index
Random Forest	CNV	Set A	0.878 (0.709–0.893)	0.720 (0.670–0.906)	0.793 (0.765–0.815)	0.587
Random Forest	CNV	Set A + Set B′	0.895 (0.886–0.916)	0.605 (0.534–0.659)	0.739 (0.710–0.769)	0.500
Random Forest	Microbiome	Set A	0.116 (0.096–0.128)	0.925 (0.892–0.958)	0.551 (0.532–0.565)	0.043
Random Forest	Microbiome	Set A + Set B′	0.058 (0.037–0.105)	0.960 (0.950–0.970)	0.543 (0.533–0.563)	0.017
Random Forest	Combined	Set A	0.872 (0.734–0.928)	0.690 (0.606–0.859)	0.780 (0.752–0.801)	0.573
Random Forest	Combined	Set A + Set B′	0.895 (0.886–0.907)	0.585 (0.465–0.658)	0.728 (0.664–0.773)	0.480
XGBoost	CNV	Set A	0.750 (0.691–0.809)	0.885 (0.855–0.916)	0.825 (0.802–0.833)	0.642
XGBoost	CNV	Set A + Set B′	0.686 (0.686–0.757)	0.975 (0.866–1.000)	0.847 (0.815–0.859)	0.671
XGBoost	Microbiome	Set A	0.099 (0.081–0.140)	0.960 (0.843–0.970)	0.556 (0.517–0.567)	0.046
XGBoost	Microbiome	Set A + Set B′	0.140 (0.084–0.158)	0.895 (0.867–0.946)	0.543 (0.523–0.569)	0.030
XGBoost	Combined	Set A	0.762 (0.668–0.797)	0.895 (0.850–0.978)	0.820 (0.796–0.881)	0.632
XGBoost	Combined	Set A + Set B′	0.703 (0.674–0.742)	0.980 (0.907–0.998)	0.844 (0.824–0.867)	0.671
GLM	CNV	Set A	0.419 (0.314–0.530)	0.470 (0.370–0.572)	0.446 (0.373–0.521)	−0.111
GLM	CNV	Set A + Set B′	0.605 (0.495–0.707)	0.460 (0.360–0.563)	0.527 (0.453–0.600)	0.065
GLM	Microbiome	Set A	0.256 (0.170–0.365)	0.740 (0.643–0.822)	0.516 (0.442–0.590)	−0.004
GLM	Microbiome	Set A + Set B′	0.442 (0.336–0.553)	0.470 (0.360–0.536)	0.457 (0.384–0.532)	−0.088
GLM	Combined	Set A	0.423 (0.317–0.534)	0.464 (0.364–0.567)	0.454 (0.381–0.529)	−0.113
GLM	Combined	Set A + Set B′	0.613 (0.503–0.714)	0.434 (0.336–0.537)	0.543 (0.469–0.616)	0.047

Set B′ denotes the filtered pseudo-labeled set.

### 3.4 Validation of XGBoost as the default implementation within MARM

To further assess the suitability of XGBoost as the default implementation of MARM, we compared the performance of XGBoost, Random Forest, and GLM under different feature-input settings, with the results summarized in Table 2. Overall, XGBoost performed best, followed by Random Forest, whereas GLM showed the weakest performance. This pattern was most evident when host-derived CNV features were used as input, suggesting that the main difference among modeling approaches lay in how effectively they exploited CNV-derived information. When trained using Set A alone, the XGBoost model based on CNV features achieved a sensitivity of 0.750, a specificity of 0.885, an accuracy of 0.825, and a Youden index of 0.642, outperforming both the Random Forest-CNV model (Youden index 0.587) and the GLM-CNV model (Youden index −0.111). Although the Random Forest-CNV model achieved a higher sensitivity (0.878), its specificity was only 0.720, substantially lower than that of the XGBoost-CNV model, resulting in inferior overall discrimination. By contrast, GLM performed poorly under the CNV feature setting and did not show meaningful predictive utility. These results indicate that, under the window-level host-derived CNV representation used in this study, XGBoost was able to maintain strong classification performance while achieving a better balance between sensitivity and specificity. XGBoost also retained a relative advantage when combined features were used as input. Under training with Set A alone, the XGBoost-combined model achieved a Youden index of 0.632, higher than that of the Random Forest-combined model (0.573) and clearly better than that of the GLM-combined model (−0.113). Under the microbiome-only feature setting, all three methods performed poorly, although XGBoost and Random Forest remained slightly better than GLM. Taken together with the findings in Section 3.2, these results indicate that the main differences among models were driven by their ability to exploit CNV features, whereas the microbiome-derived features evaluated here did not provide a stable discriminative signal. Overall, these findings support the use of XGBoost as the default implementation of MARM. Its advantage was reflected not only in overall performance metrics, but also in its compatibility with the structure of the host-derived CNV features used here. Compared with linear models, XGBoost was better able to capture nonlinear combinations of abnormalities across genomic windows; compared with Random Forest, it achieved a more favorable overall discriminative balance while maintaining strong classification performance. Thus, within the risk prediction framework proposed in this study, XGBoost represented the more appropriate default implementation.

### 3.5 Effect of pseudo-label-based extension training with weakly labeled samples on MARM performance

**Figure 3:**
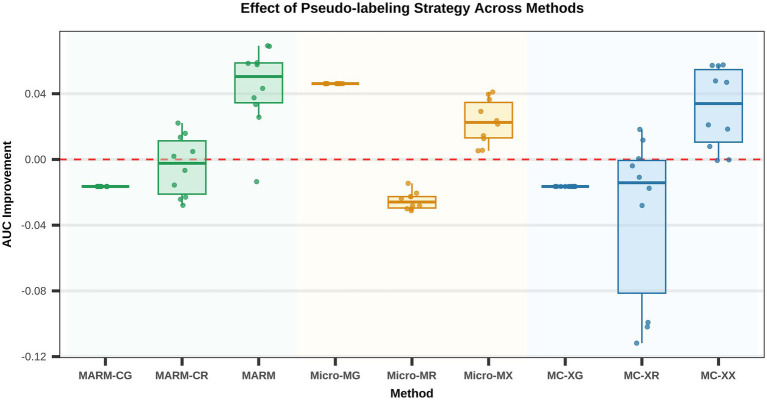
Effect of pseudo-label-based weakly labeled sample expansion on the performance of different models. The figure compares changes in AUC before and after the introduction of pseudo-labels under CNV, microbial, and combined feature settings across different machine learning methods. Different colors represent different machine learning methods.

The effect of pseudo-label-based extension training with weakly labeled samples on model performance is shown in Figure 3, with the corresponding metric changes summarized in Table 2. Overall, after incorporating pseudo-labeled samples from Set B, the different modeling approaches responded differently: models within the XGBoost framework showed a clear performance gain, whereas no stable improvement was observed for Random Forest or GLM. This indicates that the usefulness of weakly labeled samples was not universal across models, but instead depended largely on the compatibility between the default modeling approach and the underlying feature structure. Within the XGBoost framework, the clearest performance gain was observed for the core model based on host-derived CNV features. The Youden index of MARM increased from 0.642 to 0.671, and that of the combined-feature model likewise increased from 0.632 to 0.671, indicating that pseudo-label-based extension training could provide a measurable performance benefit under this framework. Notably, after pseudo-label incorporation, the sensitivity of MARM decreased from 0.750 to 0.686, whereas specificity increased from 0.885 to 0.975 and accuracy increased from 0.825 to 0.847, ultimately leading to a higher Youden index. This pattern suggests that pseudo-label-based extension training did not alter the core discriminative signal used by the model, but primarily improved its ability to exclude non-malignant samples, thereby enhancing overall classification performance. By contrast, although Random Forest showed an increase in sensitivity for some models after pseudo-label incorporation, this was offset by a more marked decline in specificity, leading to an overall reduction in the Youden index. For example, for the Random Forest-CNV model, sensitivity increased from 0.878 to 0.895, whereas specificity dropped from 0.720 to 0.605 and the Youden index fell from 0.587 to 0.500. GLM showed only limited changes after pseudo-label incorporation and remained weak overall; for example, the Youden index of the GLM-CNV model increased only from −0.111 to 0.065. These findings indicate that pseudo-label-based extension training did not produce a general improvement across models, and that its benefit was strongly model dependent. Taken together, pseudo-label-based extension training is better viewed within the present framework as an enhancement step for MARM rather than as a replacement for the core modeling pipeline. Its main role was to improve the overall discriminative performance of the model without changing the fundamental structure of MARM, namely, host-derived CNV as the core signal and XGBoost as the default implementation. We further examined whether the performance of the pseudo-label-extended MARM model was sensitive to the confidence threshold used for pseudo-label selection. Under otherwise identical workflows, thresholds of 0.7, 0.8, and 0.9 were compared on the independent validation set, Set C. The corresponding median AUC values were 0.871, 0.926, and 0.900, respectively. Among these settings, the 0.8 threshold achieved the best overall performance and showed relatively limited variability (SD = 0.0254), supporting its use as the default threshold in the main analysis. These results indicate that pseudo-label threshold selection can affect model performance, and that a threshold of 0.8 provided a reasonable balance between pseudo-label purity and effective sample expansion in the present study. The detailed comparison is provided in Supplementary Figure S3.

### 3.6 Overall performance of MARM and identification of the preferred implementation

**Figure 4:**
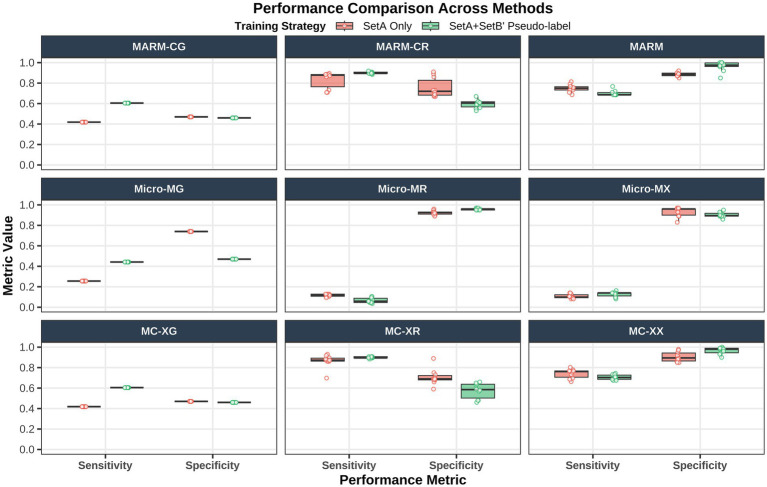
Comparison of sensitivity and specificity across different models under different feature types and training strategies. Sensitivity and specificity are shown for CNV, microbiome, and combined features under different training strategies, stratified by machine-learning method.

The distribution of sensitivity and specificity across models is shown in Figure 4, and the corresponding summary performance metrics with 95% confidence intervals are presented in Table 2. Based on the combined evaluation of sensitivity, specificity, accuracy, and the Youden index, the best-performing model was the pseudo-label-enhanced MARM, i.e., the XGBoost model built on host-derived CNV features. This model achieved a sensitivity of 0.686, a specificity of 0.975, an accuracy of 0.847, and a Youden index of 0.671. Although the XGBoost-combined model reached the same Youden index (0.671) under the Set A + Set B′ training condition, the combined-feature model did not show a clear or stable advantage over the CNV-only model. In addition, the CNV-only model was simpler in feature composition and followed a more direct methodological path, which was more consistent with the design logic of this study, namely, using host-derived CNV as the core modeling signal. Accordingly, the XGBoost-based CNV model was considered the preferred implementation of MARM. Taken together with the findings in Sections 3.2–3.4, the results support a consistent conclusion: in microbially admixed BALF mNGS data, the principal discriminative information for malignancy prediction is derived from host CNV; within this setting, XGBoost is the default modeling approach best matched to the feature structure; and pseudo-label-based extension training with weakly labeled samples can further improve overall model performance without altering the underlying source of the core discriminative signal. Overall, these results validate MARM as a malignancy prediction framework centered on host-derived CNV, implemented by default with XGBoost, and further enhanced through pseudo-label-based extension training.

### 3.7 Genomic regions contributing most strongly to MARM

To improve the biological interpretability of MARM, we further examined the top 10 CNV features with the highest contribution in the final model and mapped them to their corresponding genomic intervals. The results showed that these high-contribution features were concentrated mainly on the X chromosome and selected regions of chromosome 3, suggesting that model discrimination was not driven merely by random window-level noise, but by a subset of genomic regions with relatively stable contribution to classification. Although the present 1-Mb window-based shallow CNV representation does not support fine localization to individual driver genes, this analysis indicates that the predictive ability of MARM arises from a group of informative chromosomal regions rather than from overall CNV burden alone. The detailed feature map is shown in Supplementary Figure S4.

## 4 Discussion and conclusion

This study addressed a specific methodological problem: how to predict malignancy from microbially admixed BALF mNGS data. To this end, we developed MARM and evaluated its key components. The performance of MARM was supported by three main findings. First, host-derived CNV represented the principal and most stable discriminative signal for this task. Second, XGBoost was better matched to the structure of the window-level CNV features than the alternative modeling approaches examined. Third, pseudo-label-based extension training with weakly labeled samples further improved model performance without altering the core framework. Together, these results indicate that, within the complex BALF mNGS background containing both microbial and host-derived sequences, the most stable malignancy-associated discriminative information arises primarily from host-level chromosomal instability rather than from the species-level microbial features evaluated here. Malignancies are commonly accompanied by copy-number gains, losses, and large-scale chromosomal abnormalities, and mNGS, while used for pathogen detection, also preserves substantial host-derived sequence information, thereby enabling CNV signal extraction from the same sample. Previous studies have shown that host-CNV analysis based on mNGS can assist in detecting occult malignancies and has value in lung cancer, body-fluid tumor screening, and the differential diagnosis of infection versus malignancy (Gu et al., 2021; Guo et al., 2021; Hemmati et al., 2024). Our findings extend this observation by showing that, in BALF samples, such signals can not only be detected but can also be used for malignancy risk modeling. The distribution of top-contributing CNV features also supports the biological relevance of the model. These features were concentrated mainly on the X chromosome and selected regions of chromosome 3, indicating that the predictive signal was driven by a subset of informative genomic regions rather than by random fluctuation alone. However, because the present study used a shallow, 1-Mb window-based CNV representation, the current interpretation remains at the chromosomal-segment level, and more detailed biological explanation will require future integration with annotated cancer-related CNV events and gene-level functional information.

By contrast, microbial features did not yield stable classification performance. At the descriptive level, microbiome-related metrics did not show consistent directional changes across datasets; at the modeling level, microbiome-only models performed poorly overall, and combined models did not show a stable gain over CNV-only models. Although prior studies have suggested associations between pulmonary microbial community alterations and lung cancer (Ramírez-Labrada et al., 2020; Zheng et al., 2021; Kanteres et al., 2025), these associations did not translate into reproducible and generalizable predictive signals in the present study under the current feature representation. This indicates that, under the current data conditions and feature-construction strategy, microbial information did not provide stable additional discriminative value for malignancy prediction. However, this conclusion should be interpreted within the scope of the present study, as the microbial features used here were based primarily on species-level detection and abundance and did not incorporate other potentially informative representations, such as pathogenicity-aware categorization, functional pathway features, community-network structure, or host–microbe interaction features. Accordingly, the limited contribution of microbial features observed here should not be interpreted as evidence that microbial information lacks broader potential relevance, but rather that it did not provide reproducible additional predictive value under the present analytical framework. The failure of combined features to consistently outperform CNV-only modeling is also important from a methodological perspective. It suggests that, under the present data structure and feature design, adding species-level microbial features did not contribute stable additional discriminative value beyond host-derived CNV. Given that the principal useful information was concentrated at the host-CNV level and that the combined models did not show a clear or sustained performance advantage, a simpler modeling path centered on CNV appears more justified. Our results also indicate that pseudo-label-based extension training with weakly labeled samples can serve as an enhancement module for MARM. The benefit was observed primarily within the XGBoost framework and was not stable in Random Forest or GLM. This suggests that the value of weakly labeled samples depends on whether the model can exploit latent structure in the added samples while remaining tolerant to pseudo-label noise. In MARM, the inclusion of high-confidence pseudo-labeled samples improved the Youden index, accuracy, and specificity, while leaving both the source of the core discriminative signal and the default modeling logic unchanged. Pseudo-label extension training is therefore better viewed as an enhancement step rather than as an alternative modeling strategy. In terms of overall performance, the pseudo-label-enhanced MARM represented the preferred implementation in this study. Although the combined model reached the same Youden index under a specific condition, it did not demonstrate a stable gain and introduced additional complexity. By contrast, MARM maintained acceptable sensitivity while achieving high specificity and accuracy, resulting in a more favorable overall balance across performance metrics. Nevertheless, the stability and broader applicability of the weakly labeled sample extension strategy still require further evaluation through more rigorous internal simulation studies and larger external validation cohorts.

This study also has several boundaries. First, Set B consisted of weakly labeled samples with incomplete outcome information; although pseudo-label extension training allowed partial use of these data, it may still have introduced error. Second, the microbial features used here were based primarily on species detection and abundance and did not incorporate pathogenicity-aware stratification, such as the distinction among established pathogens, opportunistic pathogens, commensal flora, and likely environmental contaminants. This is particularly relevant for BALF samples, in which oral microbiome contamination, airway colonization, and infection-related background variation may all affect the clinical interpretation of microbial signals. Accordingly, the more precise interpretation of our findings is that microbial information did not provide stable additional value under the current feature-construction strategy, rather than that microbial information lacks potential value altogether. Accordingly, the more precise interpretation of our findings is that microbial information did not provide stable additional value under the current feature-construction strategy, rather than that microbial information lacks potential value altogether. Third, the analysis was based exclusively on BALF samples and is therefore most directly applicable to pulmonary disease settings. Fourth, although Set C provided independent validation, all datasets in the present study were derived from a relatively similar overall setting, and larger multicenter external validation remains lacking. Given that BALF mNGS data may vary across sequencing platforms, laboratory workflows, patient populations, and clinical environments, the transportability of the present model to other settings still requires further evaluation. In particular, differences in host-read fraction, sequencing depth, background microbial composition, and preprocessing pipelines may affect feature stability and model performance. Therefore, the current findings should be interpreted as evidence of feasibility within the present data setting rather than as proof of broad generalizability.

Taken together, the present study shows that, in microbially admixed BALF mNGS data, host-derived CNV is more suitable than the evaluated microbial features as the core modeling signal for malignancy prediction. This is reflected not only in its superior discriminative performance, but also in its relative stability across models and training strategies. In contrast, microbial features showed less stable directional changes and less consistent model contribution, limiting their ability to support robust prediction on their own. On this basis, MARM was constructed as a malignancy prediction model centered on host-derived CNV, providing a practical solution for malignancy prediction in complex samples and a methodological reference for related clinical auxiliary screening applications.

## Data Availability

The original contributions presented in the study are included in the article/Supplementary material, further inquiries can be directed to the corresponding authors.
