# Supplementary figures and images for: MARM: a framework for malignancy risk prediction from host-derived CNV in bronchoalveolar lavage fluid mNGS data with microbial admixture
Source: Front Microbiol. 2026 May 21;17:1846545. doi: 10.3389/fmicb.2026.1846545 (PMC13233351; doi:10.3389/fmicb.2026.1846545)

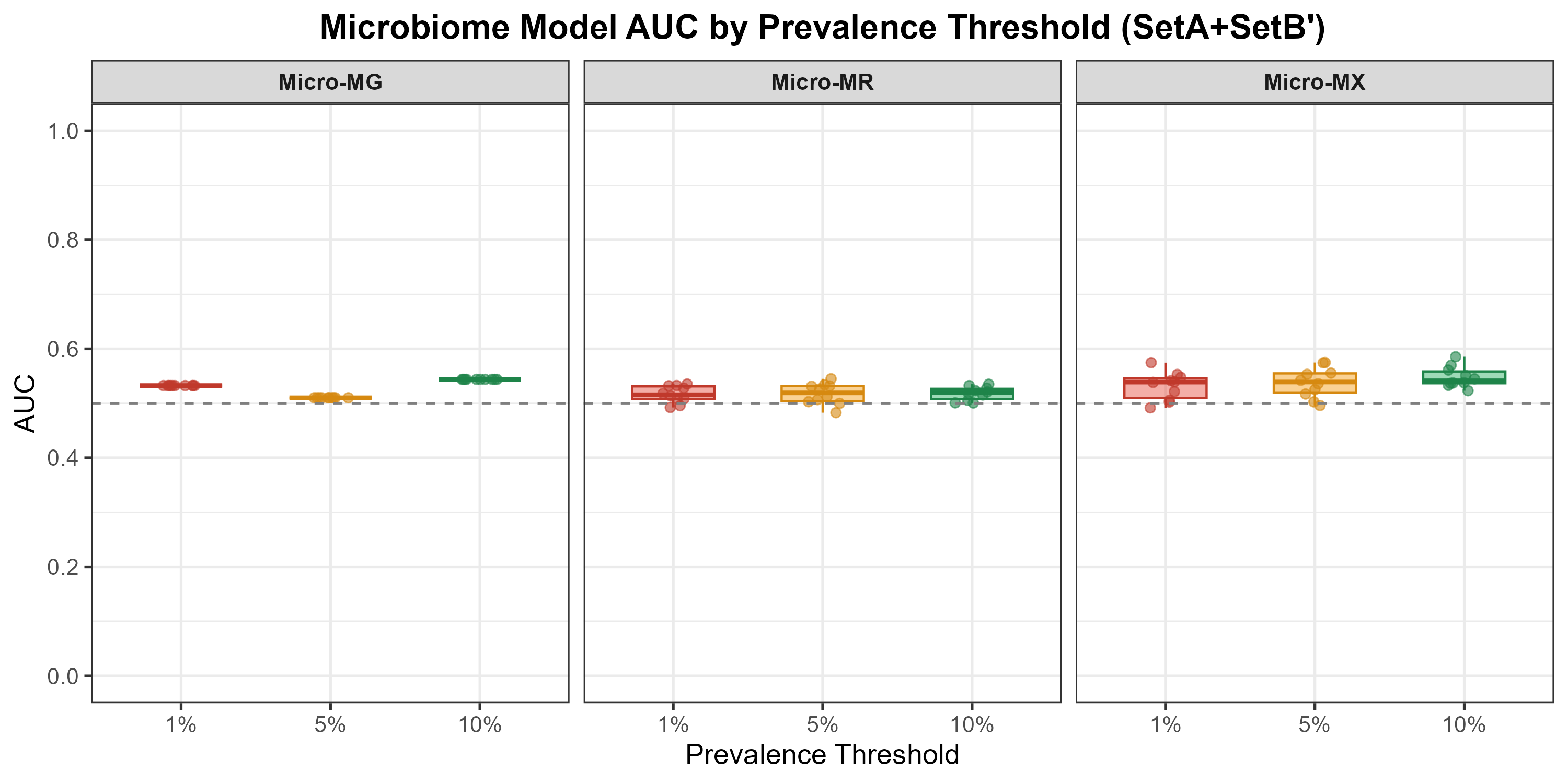

Supplement: Supplementary file 1 [file Image_1.TIFF]

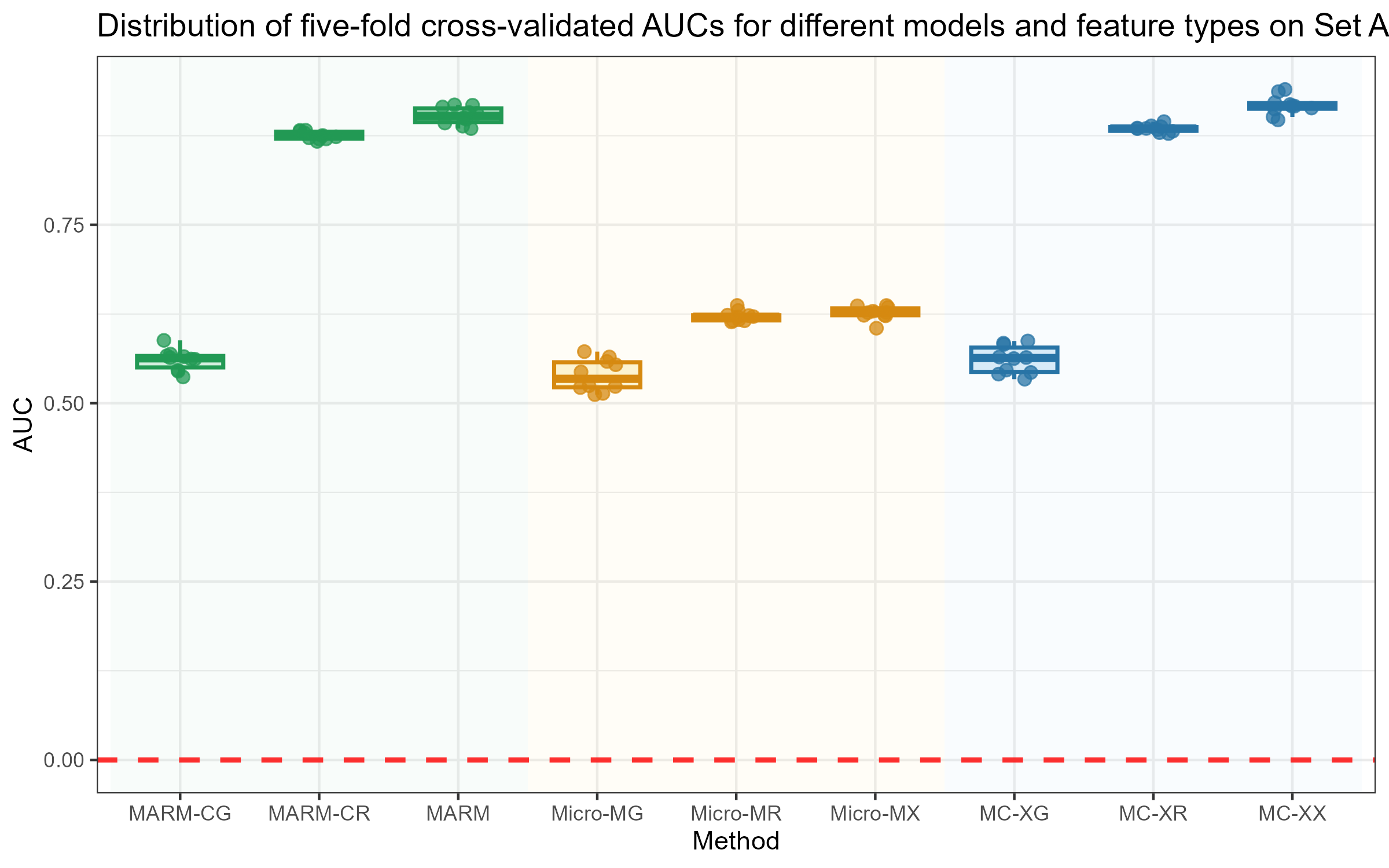

Supplement: Supplementary file 2 [file Image_2.TIFF]

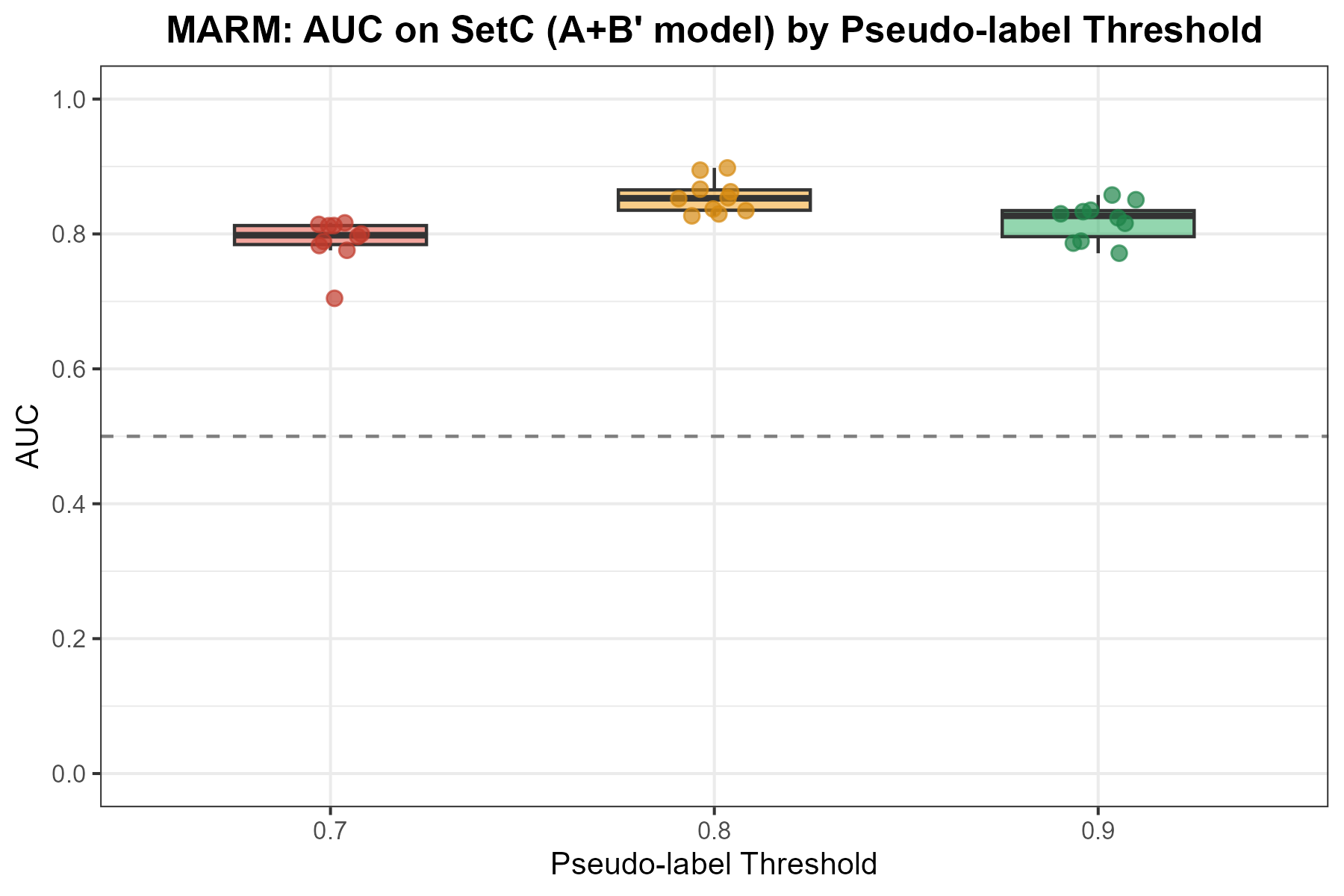

Supplement: Supplementary file 3 [file Image_3.TIFF]

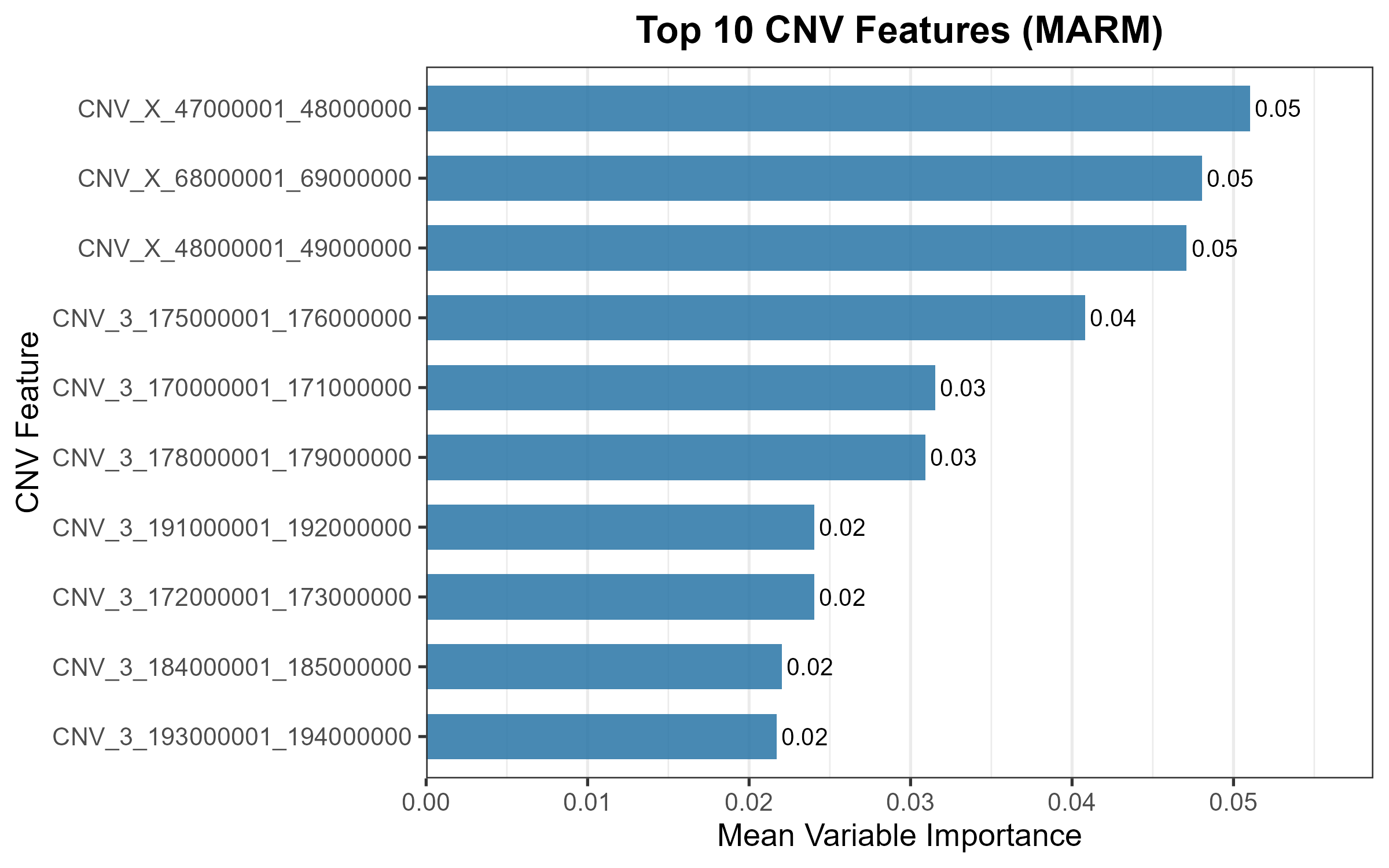

Supplement: Supplementary file 4 [file Image_4.TIFF]
